# Improving classification in protein structure databases using text mining

**DOI:** 10.1186/1471-2105-10-129

**Published:** 2009-05-05

**Authors:** Antonis Koussounadis, Oliver C Redfern, David T Jones

**Affiliations:** 1 Bioinformatics Group, Department of Computer Science, University College of London, London, WC1E 6BT, UK; 2 Department of Structural and Molecular Biology, University College of London, London, WC1E 6BT, UK

## Abstract

**Background:**

The classification of protein domains in the CATH resource is primarily based on structural comparisons, sequence similarity and manual analysis. One of the main bottlenecks in the processing of new entries is the evaluation of 'borderline' cases by human curators with reference to the literature, and better tools for helping both expert and non-expert users quickly identify relevant functional information from text are urgently needed. A text based method for protein classification is presented, which complements the existing sequence and structure-based approaches, especially in cases exhibiting low similarity to existing members and requiring manual intervention. The method is based on the assumption that textual similarity between sets of documents relating to proteins reflects biological function similarities and can be exploited to make classification decisions.

**Results:**

An optimal strategy for the text comparisons was identified by using an established gold standard enzyme dataset. Filtering of the abstracts using a machine learning approach to discriminate sentences containing functional, structural and classification information that are relevant to the protein classification task improved performance. Testing this classification scheme on a dataset of 'borderline' protein domains that lack significant sequence or structure similarity to classified proteins showed that although, as expected, the structural similarity classifiers perform better on average, there is a significant benefit in incorporating text similarity in logistic regression models, indicating significant orthogonality in this additional information. Coverage was significantly increased especially at low error rates, which is important for routine classification tasks: 15.3% for the combined structure and text classifier compared to 10% for the structural classifier alone, at 10^-3 ^error rate. Finally when only the highest scoring predictions were used to infer classification, an extra 4.2% of correct decisions were made by the combined classifier.

**Conclusion:**

We have described a simple text based method to classify protein domains that demonstrates an improvement over existing methods. The method is unique in incorporating structural and text based classifiers directly and is particularly useful in cases where inconclusive evidence from sequence or structure similarity requires laborious manual classification.

## Background

Advances in structural biology have increased the rate at which new protein structures are being determined, thus creating a need for automated methods for protein domain classification. The main computational tools for classification in protein structure databases such as CATH [1] and SCOP [2] remain sequence and structural comparisons. Indeed, in the processing of CATH, for example, a high degree of structural similarity often warrants the direct inheritance of the classification of the matched known domains. Although it may be possible to classify protein domains purely on the basis of clear sequence and structural similarity, there are many cases that exhibit 'borderline' or low similarity to existing members which require laborious manual classification. This manual classification usually requires study of the relevant literature, and so classification of these 'borderline' domains may benefit from automated literature analysis. To address this need, text mining based methods may complement the existing molecular computational approaches, especially in cases where the evidence from such sequence and structural similarities is inconclusive.

Support Vector Machines (SVM) are one of the newer machine learning approaches in wide usage today, and they are based on statistical methods to minimise the risk of error and offer solutions for optimal generalisation performance [3]. SVMs exploit statistical learning theory and are capable of overcoming the problems commonly associated with high dimensionality, such as overfitting. SVMs have been demonstrated to perform well in document classification tasks [4]. In such applications, text documents are represented as vectors according to the bag-of-words model, whereby each word represents a dimension in a high-dimensionality space. These vectors not only have high dimensionality, but they are also sparse at the same time, as each document typically contains a small subset of the very large set of words which are present in the corpus vocabulary. SVMs are based on learning a separating hyperplane that divides two sets of vectors such that the risk of misclassification is minimized, and are particularly suitable for this kind of high dimensionality, sparse data.

There are several applications of SVMs for document classification. PreBIND is an information extraction system based on SVM technology for the detection of protein-protein interactions in the literature [5]. Stapley *et al*. [6] used SVMs to infer the sub-cellular location of proteins from text sources. Rice *et al*. [7] developed a machine learning approach to mine protein function predictions from text. Another SVM based document classification algorithm was implemented to assign documents into nine main categories which correspond to chapters of the WormBook. The system was optimised for the *Caenorhabditis elegans *corpus using rules, but the same classification engine can be applied to other domains [8].

Several approaches already exist that incorporate text mining methods and other bioinformatics tools. Indeed, the combination of sensitive sequence similarity searches with functional annotations has been successfully implemented for the functional prediction of proteins, based on experimental knowledge of remote homologues [9]. Other attempts combine functional information with a variety of similarity search methods. SAWTED (Structure Assignment With Text Description) uses textual descriptions from several fields of UniProt records to enhance the detection of remote homologues from PSI-BLAST results [10]. ProFAT combines PSI-BLAST sequence similarity searches with fold recognition and text mining to refine hits according to their function [11].

Various methods are available for automatic functional annotation of proteins from text, and several of these have been evaluated in the second task of BioCreAtIvE [12]. Couto *et al*., have developed an unsupervised method for recognizing biological properties in free text (FiGO) [13]. The system splits text in sentences and considers the evidence content based on the nomenclature of a genomic ontology that structures the properties. GOAnnotator [14] is able to link Gene Ontology (GO) terms in uncurated annotations of UniProt entries with evidence text from documents related to such entries. Other methods for automatic identification of GO terms in free text include the approach by Ruch [15] which is based on pattern matching and text categorisation ranking, and by Gaudan *et al*. [16] which considers not only the presence of words of the GO terms occurring in text, but also proximity between words and their specificity based on their information content.

We have developed a novel text based approach for protein classification, which is based in text similarity of documents related to proteins, with a view to support the curation of protein structure databases. It is assumed that textual similarity between sets of documents relating to proteins mirrors structural and functional relationships and therefore can be used to make protein classification assignments. Such documents usually contain a description of the protein function and protein structure. In addition, they may mention specific characteristics, related or homologous proteins and their classification. In general, they represent concepts that report on functional and structural details. Several of the words used in these descriptions are specific to each protein or protein class. The method exploits the presence of such protein specific words (features) in document classification. It is expected that documents related to proteins that belong to the same class, and therefore have similar function and structure, are more similar than documents related to proteins in different classes. A classification assignment for a query protein can be inferred by assessing text similarity between a set of documents relating to the unclassified protein and sets of documents relating to classified proteins. For instance, if documents related to an unclassified protein are similar to documents related to a classified protein, then it could be that they belong to the same class, or in this case, the same protein superfamily.

The documents can be abstracts or full text articles, although only abstracts have been used here due to the large variety of access control methods employed on journal full texts. An SVM model was developed to discriminate sentences in the abstracts containing functional, structural and classification information that are relevant to the protein classification task. The SVM model was used to remove text that is irrelevant to the classification task, thus reducing noise and increasing the prevalence of informative terms, allowing for more accurate classification predictions. The SVM model presented is novel and optimised for the discrimination of sentences in text that contain useful information for protein classification.

The text similarity algorithm was firstly optimised using a previously described gold standard dataset [17]. Several conditions were tested, such as the inclusion of additional text from related articles and UniProt [18] and Protein Data Bank [19] annotations, as well as the filtering of the abstracts using the SVM model. The optimal conditions were then applied in text comparisons within a much larger collection of documents that relate to proteins already classified in the superfamily (H) level of the CATH database. Although the structural similarity classifier performed best, text similarity was useful in a logistic regression model that combined the two classifiers.

The method is unique in incorporating structural similarity searches with text mining directly. In a dataset of 'borderline' proteins, which lack clear structural and sequence similarity to classified proteins, the combination of the structural and text similarity classifiers resulted in an improvement in coverage by up to 50% at low error rates (10^-3^), compared to the structural classifier alone. Additionally, it makes an extra 4.2% of correct classification decisions when only the highest scoring predictions were used to infer classification. This method is useful for the challenging task of classification of such 'borderline' cases that usually require manual curation involving time-consuming study of the relevant literature.

### Application to CATH database

The CATH database is a hierarchical classification of protein domain structures in PDB. There are four major levels in this hierarchy: Class, Architecture, Topology (Fold family) and Homologous superfamily. The latter level groups together domains where evidence from sequence, structure and function similarity suggest they have evolved from a common ancestor. Classification is currently guided by structure and sequence similarity measures such as CATHEDRAL [20], SSAP [21], profile HMMs [22] and manual procedures such as literature analysis. The SSAP structure comparison method uses a double dynamic programming algorithm to align two protein structures.

In CATH, an existing classification is inherited if a protein domain displays "clear" structural and sequence similarity (over 35% sequence identity and/or SSAP score over 80) with a classified domain. However, if there is no domain in the database that fulfils these requirements, the classification is performed manually by considering the results of structural and sequence similarity and analysis of relevant literature. The literature often contains references to the function of the protein and even which evolutionary family it is thought to belong to. It is this information that can be highly useful for classification and lends itself to a text mining approach.

## Results

### Combined structure and text classifier outperforms structural similarity in protein classification of 'borderline' cases in CATH

An all-versus-all text comparison was performed using DC1.1993 as the query set and textCATH as the reference set according to the optimal conditions identified in the gold standard enzyme dataset. Performance was assessed using the AUC and MCC measures (Table 1). The structural similarity classifier SSAP performed best, while the classification power of text similarity was lower as judged from the AUC metric (0.908 and 0.789, respectively) and the MCC (0.23 and 0.12, respectively). Performance among the SVM-filtered and intact reference sets was almost identical, however the SVM filtered set (textCATH) had fewer terms (lower dimensionality). Although the text classifier performance was average, its usefulness was investigated for adding value to the better performing structural classifier in cases with low structural and sequence similarity.

**Table 1:** Performance of structure, text classifiers and logistic regression models in 'borderline' proteins from CATH

	**DC1.1993^a^**		**Training set^b^**			**Test Set^c^**	
	
**CLASSIFIER**	**AUC**	**MCC**	**AUC**	**R**^2^	**Model LR**	**AUC**	**MCC**
SSAP + TEXT	0.920	0.29	0.924	0.34	70021	0.917	0.28

SSAP	0.908	0.23	0.913	0.30	62182	0.905	0.22

TEXT	0.789	0.12	0.791	0.06	11814	0.788	0.12

The performance of the structural similarity measured by the SSAP algorithm and text similarity (TEXT) as classifiers for protein classification in the homologous superfamily level in the DC1.1993 dataset of 'borderline' cases in CATH using the textCATH as reference set. Classification performance was assessed using the AUC and MCC measures on the whole set (a), training (b), and test (c) sets. Nagelkerke's R^2 ^is a measure of the variance accounted by the variables of the logistic regression models. Model L.R. stands for model likelihood chi-square which is the difference between Null and Residual deviance. Logistic regression models were trained on a random subset of comparisons from 1000 abstracts and tested on the remaining 993 abstracts from the 'borderline' cases dataset DC1.1993.

To check if it is possible to develop an optimal combination of the classifiers (SSAP and text similarity) by exploiting any orthogonality in our data, we generated a series of logistic regression models. The inclusion of the text similarity (TEXT) variable was significant (p <= 0.001) as judged from the increase in the likelihood ratio which reflects the difference between error not knowing the independent variables and error when the independents are included in the model (Table 1). The importance of the explanatory variables was also confirmed by carrying out Wald chi-square tests for statistical significance (Table 2). In the model that included the structure and text similarity, both explanatory variables contributed significantly to the model effect, but the structural similarity classifier SSAP accounted for the largest effect among the independents. The equation coefficients of the model (SSAP+TEXT) are shown in Table 2.

**Table 2:** Coefficients and Wald tests for logistic regression model

	**Coeff**	**S.E.**	**Wald Z**	**P**
Intercept	-23.4406	0.093	-251.91	< 0.001

SSAP	0.2891	0.001	211.59	< 0.001

TEXT	0.1254	0.001	96.58	< 0.001

Coefficients and Wald Z statistics of the logistic regression model SSAP+TEXT that includes SSAP (structural similarity) and TEXT (text similarity) independent variables. Coeff = coefficient for the logistic regression; S.E. = standard error; Wald Z, p = Wald statistic with corresponding probability.

**Figure 1:**
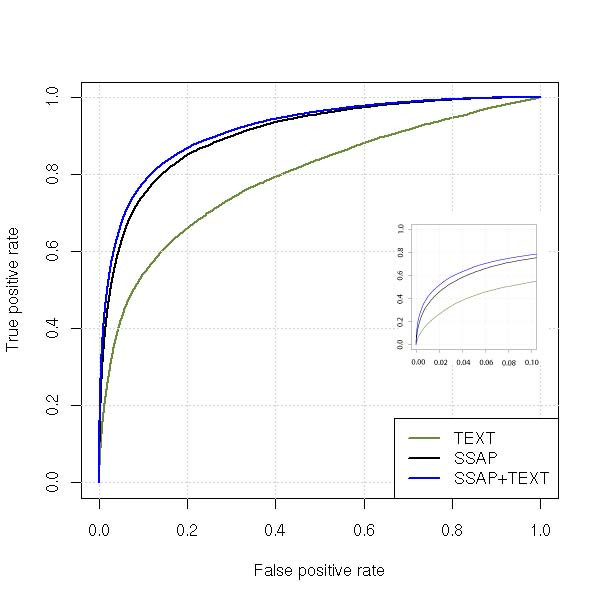
**Test ROC curves of the text similarity algorithm in the 'borderline' cases dataset**. ROC curves of the test set from the 'borderline' cases DC1.1993 dataset for the TEXT (green), SSAP (black) classifiers and the logistic regression model that includes SSAP and TEXT independent variables (blue). The reference set was textCATH. The inset shows the same curves for low error rates (FPR < 0.10).

The discriminative power of the models (how well matches are distinguished from non-matches) was evaluated using the AUC statistic. Its value is the total fraction of cases where a classification match is ranked higher than a non-match. Values of AUC for models that contain structural classifier SSAP are already over 0.90 indicating significant extant predictive capacity from structure alone. In contrast, the AUC for the text similarity classifier alone was only 0.79, thus indicating lower classification power when textual information is considered in isolation. Nevertheless, this indicates that the text classification pipeline is extracting almost 88% of the structural information when assessed against the opinion of an expert curator. Clearly highly relevant texts are being extracted, which would be useful to a human curator in the intended practical application. Interestingly, inclusion of the TEXT variable to the structural classifier improves the AUC value to 0.91 for the combined SSAP and TEXT model (Figure 1, Table 1). This clearly indicates that the text classifier not only provides consistent support for the structure-based classification, but also increases coverage. Classifier accuracy was further analysed using the Matthews Correlation Coefficient (MCC). The results indicated that the combined model SSAP+TEXT outperformed the SSAP structural classifier (Table 1). The significance of the improvements in the MCC was evaluated using Fisher's Z test, which considers the magnitude of the difference and the strength of the correlation. The improvements in MCC values were statistically significant (p < 0.001). Further, the combined SSAP and TEXT model R^2 ^was improved (0.34) compared to SSAP (0.30), indicating that the model is useful in classification predictions (Table 1). These results demonstrated the real improvement in performance by the inclusion of TEXT variable in the combined logistic regression models.

### Comparative classifier performance in protein classification

**Figure 2:**
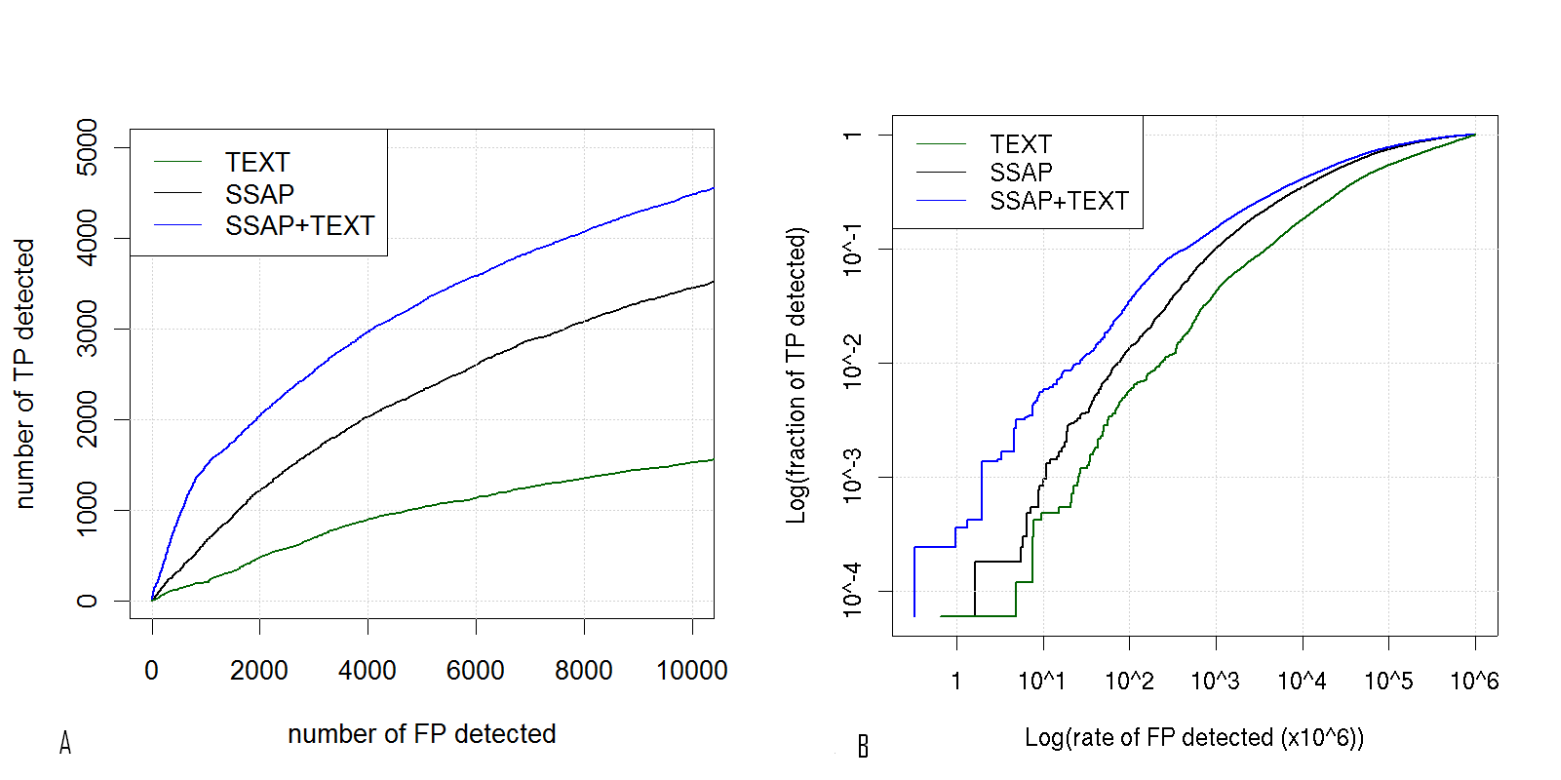
**Coverage versus error graphs**. (i) Coverage (sensitivity) versus error graph. For each classifier, the scores of the comparisons between the query DC1.1993 'borderline' set and the reference textCATH set were sorted in decreasing order. The comparisons include both CATH superfamily classification matches (true positives TP) and non-matches (false positives FP). Descending from the top classifier score, the numbers of true and false positives are counted for each possible cutoff. Green: TEXT; black: SSAP; blue: SSAP + TEXT logistic regression model. (ii) Log of the fraction of true positives versus the log of the false positive rate (FPR) graph. The FPR is defined as the fraction of the total false positives for each score cutoff. The fraction of TP is the proportion of the total number of TPs (see text).

In order to compare the performance of the SSAP, TEXT and SSAP+TEXT classifiers "coverage versus error" plots were used [23]. The plots were constructed by collecting all comparison scores for each classifier and ranking them in reverse order. Moving down the list, the numbers of matches (true positives) accumulated thus far were counted and plotted versus the number of non-matches (false positives) for each score cutoff (Figure 2A). For each classifier, the fraction of the total number of matches or coverage, was also calculated at certain error rates. The fraction of the total number of matches is the number of true positives divided by the total number of true matches (16765) in the test set.

The false positive rate is the fraction of non-matches for a score cutoff. For instance, FPR of 10^-6 ^indicates that the classifier makes 3 erroneous predictions out of the possible 3059841. Likewise, FPR of 10^-5^, 10^-4 ^and 10^-3 ^correspond to 31, 306, 3060 and 30598 errors, respectively. Table 3 shows the matches detected as an actual number and as a percentage of the total matches, for a range of FPR values. The results are shown graphically in Figure 2B.

**Table 3:** Number of classification matches at various rates of false positives in the 'borderline' DC1.1993 dataset

Errors		CATH superfamily classification matches (TP)					
		TEXT		SSAP		SSAP + TEXT	
		
False Positive Rate	Number of errors	Coverage	Cutoff	Coverage	Cutoff	Coverage	Cutoff

10^-5^	31	8; 0.04	77.70	16; 0.09	79.94	98; 0.58	0.9808

10^-4^	306	96; 0.57	48.86	229; 1.36	79.40	585; 3.48	0.6792

10^-3^	3060	707; 4.21	20.75	1677; 10.00	76.66	2571; 15.33	0.2982

10^-2^	30598	3036; 18.10	7.83	5808; 34.64	71.24	6901; 41.16	0.0706

Coverage is the fraction of true classification matches and is shown as actual numbers and as a percentage of total TP (%). Scores range between 1 and 100, 30 and 80, and 0 and 1 for the TEXT, SSAP and SSAP + TEXT classifiers, respectively. Total comparisons: 3076606, positive matches: 16765.

The data in Figures 2A and 2B and Table 3 demonstrate that at low false positive rates, 10^-6 ^and 10^-5^, all classifiers display low coverage. At a higher rate of false positives (10^-3^), the SSAP, TEXT and SSAP+TEXT classifiers detect 10%, 4.21% and 15.33% of the true matches. The SSAP+TEXT classifier consistently outperforms the structural classifier when used on its own, especially in the range of low error, with more than double the number of matches found for error rates below 10^-4^. The TP-FP breakeven point is the number of true positives that equals the number of false positives. In the test set for the SSAP+TEXT, the breakeven point is reached after 2098 matches are detected (12.51%). Both SSAP and TEXT predict more false than true positives for all cutoffs in the 'borderline' dataset.

Aside from the ability of each classifier to separate true from false positives across the range of scores, we were also interested in the power of each method to assign correct structural classifications to proteins of the 'borderline' set in CATH using the top scoring hit. Only the best scoring domain comparison was considered for each of the 2436 domains of the DC1.1993 set (tophit), in order to simulate classification tasks. The combined SSAP+TEXT classifier increased coverage by 4.2%, capturing 1694 (69.5%) correct classifications relative to 1591 (65.3%) by SSAP alone.

## Discussion

Our results demonstrate that although much work is required to address fully-automated classification of proteins that lack clear structural or sequence similarity, the combination of text and structure similarity can improve classification performance for 'borderline' cases. When text and structural similarity scores were compared for their classification power in a set of simulated 'borderline' cases of the CATH protein structure database, structural similarity scores performed best in the H (superfamily) level of CATH. This may be expected for structural databases, where structural relationships primarily define class membership. Although the SSAP scores perform better than text similarity in classification as single predictors, the performance improved considerably when structure and text similarity classifiers were combined in a logistic regression model.

On a dataset of 'borderline' cases, a significant improvement in coverage by up to 50% at low error rates was observed. For equivalent coverage, the combined classifier benefits from a significant reduction in the number of false positives compared to SSAP. For instance, when SSAP captures 1000 true matches, it also includes 1612 false positives, while the SSAP+TEXT classifier includes 559, which is a 65% reduction. Moreover, the combined classifier was able to make an additional 4.3% of correct classification predictions based only on the top scoring hits, compared to SSAP alone. The combined classifier is therefore a better starting point than SSAP on its own, when classifying a 'borderline' protein. Although the error rate is not sufficiently low to enable fully automatic protein classification of 'borderline' cases, the combined classifier can have practical application in the support and evaluation of manual protein domain classification assignments. More development of the text similarity algorithm using a more elaborate weighting scheme combined with more efficient text retrieval and information extraction is required to improve performance further.

The text similarity algorithm was benchmarked and optimised using a manually curated dataset of enzyme superfamilies, before it was applied on a large dataset of 'borderline' cases in CATH. Removal of sentences which were semantically irrelevant to protein classification using an SVM model resulted in better performance of the text classifier, while the dimensionality of the feature space of the document reference set of CATH was reduced by 32.7% making calculations speedier. Although all terms were used in this implementation, performance may improve further with feature selection based on thresholding metrics, such as document frequency, information gain, mutual information or chi-square and more involved weighting schemes. For example, the weights of a specific set of highly informative terms can be boosted. For the CATH database reference set, these terms would include selected names and synonyms of the superfamily and their protein members and function annotations from UniProt. Performance should also benefit by using a more sophisticated term generation scheme that accounts for synonymy.

All words of the abstracts, including title and authors, were used in our method. Typically abstracts contain a brief description of the protein structure. A more detailed and extensive description of important features may only be mentioned in the main article, so full text is expected to yield better results. Irrelevant sentences can be removed by classifying each sentence of the full text article using the SVM filter. The inclusion of additional abstracts from related articles in the text related to each protein of the reference set improved performance. Abstracts which are related to the main reference usually contain additional terms that are likely to be characteristic of each class. As full text articles are not always available in public databases, this is an alternative way to muster additional relevant text for each protein and leverage the lack of full text.

A more comprehensive selection of texts by inclusion of abstracts of relevant articles sourced from UniProt and GenBank records may provide more comprehensive text sources for the query proteins. Annotations from PDB and UniProt may also be used alternatively or additionally to abstracts in the query proteins. In this implementation, abstracts were used as text relating to the query protein of the test set because they are more readily available. Moreover, it is common for annotations to contain technical terms (for instance '3D structure'), which may introduce bias to the text similarity classifier. They also lack consistency and reliability because not all proteins are fully annotated yet, while several annotations are propagated based on high homology rather than experimental observations.

Structural database entries are protein domains, however domains lack a one-to-one relationship to PubMed abstracts. In fact, it is assumed that each abstract possesses information in all domains of its related protein. In reality it is quite frequent for certain domains within a protein to attract greater interest, and text, than others. Inclusion of additional abstracts or full text articles related to the query protein will increase the likelihood that information relevant to all domains of the protein is present in the text.

Combination of the text similarity algorithm with other structural similarity algorithms, such as CE [24], DALI [25] and MSDFold [26] via logistic regression or machine learning based methods, may provide a useful performance benefit. The same methodology, with little alteration, can also be used to improve fold recognition and structure prediction results. Moreover, the text similarity algorithm can be proven valuable in protein classification tasks where a more accurate function classifier is not available. For example, it may be useful in enzyme classification. In the Enzyme Commission classification system, enzymes are classified according to the chemical reaction they catalyse. It is likely that text similarity may be a more appropriate classifier than structure or sequence similarity for this database.

Finally, we demonstrated the practical application of a text based classifier in protein structure database curation. The model resulting from its combination with the structural classifier is superior to the structural classifier alone, thus providing an improved way to classify 'borderline' proteins in the CATH protein structure database. Although in the context of full automation the improvements might at first sight look relatively moderate, it is important to bear in mind that in the principal application domain, the textual results are intended to be used by manual curators or users of a structure classification server as a guide to manual classification. Putting it another way, we need the textual results to be confirmatory of the structural similarity results rather than being entirely novel. The fact that our text classification scheme reproduces around 88% of the purely structure-based AUC, and in combination increases the AUC by a small but significant amount, shows that we are indeed extracting the most relevant texts and that some of these texts are key to making better informed decisions on superfamily membership. A user of a hybrid system would therefore be provided with a highly relevant shortlist of texts from which he or she can make an informed decision as to the correct superfamily classification of the protein being analysed. In other words, the fact that we can improve automatic classification is actually far less important that the fact that we are able to select the relevant texts which can be further analysed by the user in semi-automatic classification. Consequently, we are planning to build this text classification functionality into the user interfaces of our existing fold recognition web servers: both the sequence-based GenTHREADER [27], and the structure-based CATHEDRAL server [28].

Overall, our findings show a useful combination of a structural similarity with a text mining approach and demonstrate the value of the text based approaches in protein classification.

## Conclusion

In summary, a text based classifier was developed and implemented for the classification of proteins in the CATH database. Although the structural similarity scores perform better than text in classification of proteins in structure databases, it was proven that the combination of the structure and text classifiers in a logistic regression model provides a more powerful classifier, significantly increasing coverage especially at low error levels compared to using structural similarity alone. The benefit is particularly useful in cases where structural similarity is not high enough to be conclusive.

We found that, for 'borderline' matches with SSAP scores below 80, which are notoriously difficult to classify, it is preferable to use the combined structure and text similarity classifier than SSAP alone. This result should be useful in the development of servers which aim to classify proteins automatically and reliably.

## Methods

### Text similarity algorithm

Text was represented according to the bag-of-words model [29] as an unordered collection of words or terms. Each document is represented as a vector of weights of the terms contained in it, as is typically for information retrieval. As for most text retrieval applications, the entries in the vector are weighted to reflect the frequency of terms in the documents and the distribution of terms across the collection as a whole. Each vector element corresponds to the frequency of each term (*TF*) in the document, weighted by the inverse document frequency of the term (*IDF*) in the document collection. *IDF *is defined as follows:

(1)

where *N *denotes the number of documents (abstracts) in the collection and *DF *is the document frequency of the term. The vectors were of unit length (L2 normalised) to compensate for variable document length which may favour long documents in the text similarity calculations. The base of the logarithm used in calculations is 10. Similarity *c *between two documents is defined as the cosine of the angle between two vectors *v *^*A *^and *v *^*B *^representing each text:

(2)

The value of *c *is high when the compared documents share several rare words. For ease of calculations, the range of c was transformed to range 0–100.

### Resources

Lucene [30]: Lucene is a high performance, scalable search engine library written entirely in Java. It is available as open source software under the Apache License and offers powerful features including text indexing, searching and term vector generation. It features a selection of analysers, including the Simple, Standard and Stop analysers which were used in this project. Analysers vary in the way they generate terms from text, removal of stop words, lowercasing, etc.

R [31]: Plotting of ROC curves, calculations of AUC, F-measure and Matthews correlation coefficient were performed using R's ROCR library [32]. Logistic regression models were generated using the Design package for R [33].

### Performance measures

The performance of the method was assessed using the AUC (area under ROC curve) metric, the Matthews correlation coefficient (MCC), F-measure and coverage versus error plots. The ROC plots were generated by sorting the scores and plotting the number of correct assignments (true positive rate) versus the fraction of erroneous assignments (false positive rate). For TP = true positives, TN = true negatives, FP = false positives, FN = false negatives, the recall (rec), precision (prec), MCC and F measure are defined as follows:









MCC is a measure of overall classifier accuracy. A value of 0 indicates random performance, whilst a value of 1 implies perfect classification. The F-measure is the weighted harmonic mean of precision (prec) and recall (rec). For α = 0.5, which is the value of α used in the assessment of this method, the mean is balanced.

### Datasets: REGS352 and PDB145

Brown *et al*. [17] describe a gold standard set of enzyme superfamilies, clustered according to sequence, structure and function, supported by references for each sequence, for use in validation of family and superfamily clustering methods. The dataset of MEDLINE abstracts REGS352 was assembled by retrieving the corresponding abstracts of the original gold set of enzyme sequences after filtering out sequences with references to personal communications or to non-sequence specific references (for example to the reference of the SWISSPROT database) [34] and sequences with irretrievable abstracts (for example papers without a PMID or a published abstract). The abstracts were classified in 5 superfamilies (classes) and in 87 families, according to their protein superfamily and family classifications (Table 4).

**Table 4:** REGS352 and PDB145 datasets

**Superfamily**	**Sequences**	**REGS352 Families**	**REGS352 References**	**PDB145 Families**	**PDB145 References**
Amidohydrolase	87	26	73	11	41

Crotonase	50	16	36	7	14

Enolase	85	9	66	8	39

Haloacid Dehalogenase	104	19	93	10	30

VOC	95	17	84	7	21

TOTAL	421	87	352	43	145

Distribution of the gold dataset sequences and the derived datasets REGS352 and PDB145 among the five superfamilies of the gold dataset. (Brown *et al *., 2006)

Another set of abstracts (PDB145) was derived from the primary references (JRNL field) of the PDB structures that correspond to the REGS352 sequences. The primary references of the 282 PDB files described in the gold dataset of enzyme superfamilies were checked in order to remove PDB files without references published in MEDLINE, and files with identical references to avoid duplicate references appearing in the set. The filtered set consists of 145 PDB files, each with a unique reference, and their distribution across the enzyme families and superfamilies of the gold set is shown in Table 4.

### Document retrieval and analysis

Abstracts relating to a protein were downloaded from PubMed using a set of Perl scripts. The title and authors with their affiliations were also collected with the body of the abstract. The primary reference for each protein was either specified by a PMID contained in the corresponding Genbank or UniProt annotation, or, if a structure for the protein was available, in the JRNL field of the relevant PDB file. Additional abstracts were collected from the 'Related Articles' hyperlink of PubMed.

To perform text comparisons, the documents were converted into IDF weighted term vectors using one of Lucene's analysers. The cosine similarity of the angle between the normalised text vectors was used to assess the similarity between text documents and was calculated according to equation (2).

### Using machine learning to screen for informative sentences

To improve performance by reducing noise and increasing the prevalence of informative terms, a machine learning approach was developed to discriminate informative sentences from any text irrelevant to the classification task within the abstracts of the reference set. Sentences from the abstracts of the REGS352 dataset were used to train an inductive linear SVM model using SVM-Light [35] for the identification of sentences with structural, functional and classification information, useful for the protein classification task. Perl scripts and regular expressions were applied to split the abstracts of the REGS352 dataset into sentences. The model was trained upon 2541 examples (1734 positive and 807 negative sentences) which have been manually classified by an expert biologist for their relevance to the protein classification task. Examples of typical informative sentences from the training set and test sentences with their SVM scores are shown in Tables 5 and 6. Typically, sentences that contained information on protein structure and function including description of specific features, on classification or in related or homologous proteins were classified as informative. Sentences containing methodological, experimental, or physicochemical data were classified as not-informative. Informative sentences are expected to contain a much higher content in words that are likely to be class specific and important in document categorisation.

**Table 5:** Example sentences used in training of the SVM model

**Training example**	**Label (+/-)**
Sequence analysis showed that *pENO2 *shares 75.6% nucleotide and 89.5% deduced amino acid sequence identity with *pENO1 *and is encoded by a distinct gene.	+

The packing of the octameric enzyme in this crystal form is unusual, because the asymmetric unit contains three subunits.	+

Cys-592, which is essential for enzymatic activity, is located in the above-mentioned histidine-rich region.	+

From the significant sequence similarity between *intradiol *enzymes, it has been shown that *intradiol *enzymes evolved from a common ancestor.	+

Two *2,3-dihydroxybiphenyl (23DHBP) dioxygenase *genes, *bphC1 *and *etbC *involved in the degradation of polychlorinated biphenyl(s) (PCBs) have been isolated and characterized from a strong PCB degrader, Rhodococcus sp.	+

A thermostable *hydantoinase *of Bacillus stearothermophilus NS1122A: cloning, sequencing, and high expression of the enzyme gene, and some properties of the expressed enzyme.	-

A *catechol 2,3-dioxygenase *gene in chromosomal DNA of P. putida KF715 was cloned and its nucleotide sequence analyzed.	-

The K+ ion activates the enzyme 100-fold with an activation constant of 6 mM, well below the physiologic concentration of K+ in E. coli.	-

A putative regulator and its possible recognition site was suggested on the basis of homology data.	-

The enzyme has a subunit Mr of 33,500 +/- 2000 by SDS/polyacrylamide-gel electrophoresis.	-

Sample positive and negative sentences manually classified by an expert biologist for their content on functional, structural and classification information and used as training examples to learn an SVM model. Terms in italics were removed prior to training.

**Table 6:** Example sentences used in testing of the SVM model

**Test sentence**	**SVM score**
These homologous proteins, designated the "enolase superfamily", include enolase as well as more metabolically specialized enzymes: mandelate racemase, galactonate dehydratase, glucarate dehydratase, muconate-lactonizing enzymes, N-acylamino acid racemase, beta-methylaspartate ammonia-lyase, and o-succinylbenzoate synthase.	3.99

GlucD is a member of the mandelate racemase (MR) subgroup of the enolase superfamily, the members of which catalyze reactions that are initiated by abstraction of the alpha-proton of a carboxylate anion substrate.	3.42

The structure of Neurospora crassa 3-carboxy-cis, cis-muconate lactonizing enzyme, a beta propeller cycloisomerase.	1.41

The corresponding cDNA was amplified from a library of lobster muscle cDNA, and a sequence corresponding to residues 27–398 was determined.	-1.10

The values for kcat were reduced 4.5 × 10(3)-fold for (R)-^®^delate and 2.9 × 10(4)-fold for (S)-mandelate; the values for kcat/Km were reduced 3 × 10(4)-fold.	-3.31

Sample positive and negative test examples classified and scored by the SVM model.

Protein, family and superfamily names were removed from the positive and negative examples to avoid unfavourable training of the model on these terms. SVMs were used to learn the features of the training set and classify new unseen sentences. Binary and frequency feature representations as well as a range of values of the c parameter of SVMlight from 0.1 to 2 were tested to identify the optimal SVM model. The frequency representation gave better results and was selected over binary. The optimal c value in our dataset was 0.25. Leave-one-out cross validation of the optimal SVM model estimated the recall 83.45% and precision 82.88%. The SVM model was used to classify each sentence in the abstracts as relevant or not-relevant. Only relevant sentences were used in the text comparisons.

### Text similarity algorithm optimisation

To benchmark the method, the text similarity scores of the abstracts of the REGS352 (test set) and PDB145 (reference set) were calculated (all-versus-all) and assessed for the prediction of classification matches in the superfamily level of CATH. Scores were labelled as matches if the CATH superfamilies of the proteins corresponding to the two abstracts were matching, or non-matches otherwise. In order to optimize the performance of the classification method several conditions of the reference set were considered and tested (Table 7).

**Table 7:** Classifier performance in the enzyme dataset

**CONDITIONS**				**PERFORMANCE**		
				**Superfamily Classification** **N = 352, 5 classes**		
				
	**Test Set**	**Reference Set**	**Lucene Analyser**	**AUC**	**MCC**	**F**

1	Abstract	Dp20 – DX33 -Ann	Stop	0.75	0.51	0.56

2	Annotations	Dp20 – DX33 -Ann	Stop	0.77	0.53	0.58

3	Abstract	Dp20 – Ann	Stop	0.74	0.50	0.53

4	Abstract	Dp20 – Ann	Standard	0.70	0.33	0.44

5	Abstract	Dp20	Stop	0.74	0.49	0.52

6	Abstract	Dp1	Stop	0.64	0.31	0.40

Classifier performance was assessed using AUC, MCC and F-measure under six conditions of the reference set PDB145: Inclusion of additional abstracts from related articles (Dp20); inclusion of annotations (Ann); filtering using the SVM model (DX33). *Conditions 1 and 2 *: 20 SVM filtered abstracts per enzyme, Stop analyser, inclusion of PDB/UniProt annotations.*Condition 3 *: 20 abstracts per enzyme, Stop analyser, inclusion of PDB/UniProt annotations. *Condition 4 *: 20 abstracts per enzyme, Standard analyser, inclusion of PDB/UniProt annotations. *Condition 5 *: 20 abstracts per enzyme, Stop analyser. *Condition 6 *: 1 abstract per enzyme, Stop analyser. For the Superfamily classification task all 352 enzymes of REGS352 were classified in 5 superfamilies. Abstracts were used in the test set, except of condition 2 were annotations from UniProt and PDB fields were used for comparison.

#### (1) SVM filtering

The SVM model was used to filter the abstracts of the PDB145 reference set for informative sentences. The SVM filtered reference set performed better (F measure: 0.56) than the set of intact abstracts (0.53).

#### (2) Retrieval of related abstracts

Perl scripts were implemented to download 19 additional abstracts for each primary reference from the 'Related Articles' hyperlink of PubMed. This function of PubMed identifies other publications in MEDLINE which resemble to the primary reference. Retrieval is based on the evaluation of similarity between two documents over all vocabulary terms using a probabilistic model [36]. The inclusion of 19 related articles from PubMed to the text related to each protein of the reference set improved the performance, presumably as a result of the inclusion of additional class-specific words which are present in the related abstracts. (AUC 0.74 for 20 abstracts versus 0.64 for 1 abstract).

#### (3) Text analysis

Three types of text analysis were tested using Lucene's Standard, Stop and Simple analysers. All analysers lowercase text, however only the first two remove stop words, and differ in the way they split the text into terms. The Standard has rules for acronyms, hostnames, etc. which are retained intact as a single term, while the Stop and Simple analysers split the words in special characters. The Standard analysis produces a large number of uniquely found, non-informative terms compared to the shorter, fewer and more abundant terms generated by the Stop analyser. Performance was improved when words were split in special characters as well as white space (AUC 0.74) instead of splitting in white space only (0.70). It also benefits from removal of 'stop' words, common words that occur in similar frequency across the reference set. There was no improvement when the Porter stemming algorithm was applied (results not shown) [37]. However, other stemmers such as the Krovetz stemmer [38] may be more suitable.

#### (4) Inclusion of annotations

A set of Perl scripts was used to retrieve annotations from the PDB (Title, Classification fields) and UniProt (Protein names, Synonyms, Function, Keywords fields) databases and concatenate them to the abstracts of the reference set. The inclusion of annotations from the UniProt and PDB databases produces a negligible improvement over the performance of the plain 20 abstracts (MCC 0.51 with annotations versus 0.50 without). The method was also tested using UniProt database annotations instead of abstracts in the test set. When relevant annotations from UniProt (Protein names, Synonyms, Function, Keywords fields) were used instead of abstracts as text relating to the query proteins, performance was improved (F measure 0.58, versus 0.56 for abstracts). However, abstracts were used in this implementation because annotations are not immediately available in the databases, in contrast to abstracts that usually accompany structural biology papers describing protein structures upon their release.

**Figure 3:**
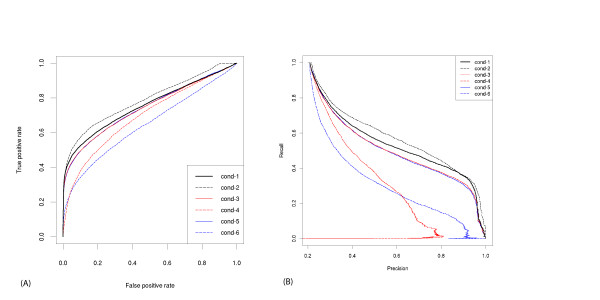
**ROC curves and precision-recall graphs of the text similarity algorithm in the enzyme dataset**. (A) ROC curves and (B) Precision-Recall graphs of conditions 1–6 (Table 7) in the enzyme dataset. Condition 1, black solid line; Condition 2, black dashed line; Condition 3, red solid line; Condition 4, red dashed line; Condition 5, blue solid line; Condition 6, blue dashed line.

The classification performance of each of the conditions is shown in Table 7. Condition 1 (20 SVM-filtered abstracts per protein, Lucene Stop analyser, inclusion of PDB/UniProt annotations) was identified as the best performer (Figure 3) and was used in text similarity calculations in CATH.

### CATH abstracts retrieval

A reference set of abstracts was assembled to assess the ability of the method to classify domains at the homologous superfamily level of CATH. CATH v3.1 contains structural classifications for domains from 30028 PDB files. Using PubMed IDs (PMIDs) indicated in the JRNL field of these PDB files of protein domains already classified at the superfamily level of CATH, a total of 15154 unique abstracts with their related articles from PubMed and annotations from UniProt and PDB were downloaded, analysed, indexed and filtered for informative sentences using the SVM model. There are 27069 PDB files (90.1%) with primary abstracts within this document set (textCATH set), because several PDB files share the same primary reference. The textCATH set included 139171 terms and covered 1806 of the 2090 superfamilies in CATH. The textCATH reference corpus is available for download from our website .

An additional reference set was compiled from the intact abstracts, without any filtering, in order to compare the effect of using the SVM model in the performance of the system. The latter set was also analysed using the Stop analyser and contained 204904 terms.

### 'Borderline' cases dataset

In order to compare the performance of the structure and text similarity measures in protein classification in the superfamily level of CATH, an all-versus-all structural comparison using SSAP was performed on the protein domains corresponding to the 15154 abstracts of the textCATH set. The majority of the protein domains have "clear" homologues in the database displaying clear structural similarity (SSAP > 80). In the benchmark, the 'easy' pairs are ignored in order to focus on protein domains without clear homologues. Only domains whose matches in the database displayed SSAP scores below 80 and sequence identity below 30% were retained in order to simulate 'borderline' classification assignments. As a result, there were 2436 'borderline' protein domains with a total of 6207493 comparisons, of which 33577 were true matches (0.54%). The set of 1993 unique primary references contained in the PDB files of such domains comprise the DC1.1993 set.

### Logistic Regression

Logistic Regression is used for prediction of the probability of occurrence of an event by fitting data to a logistic curve. The logistic model can be written as follows:



where p is the probability of a classification match, and x_1_, x_2_,..., x_i _are the explanatory, independent variables, which in this case are the SSAP structural similarity and the text similarity (TEXT) scores. The resulting score is thus defined as the natural logarithm of the odds.

For validation purposes, the set of comparisons was split into a training set using the comparisons of 1000 randomly selected abstracts and a test set comprising the comparisons of the remaining 993 abstracts. The training and test sets included 1224 and 1212 domains from the DC1.1993 'borderline' cases dataset, respectively. There were 3130887 (3076606) domain pair comparisons in the training (test) set, of which 16812 (16765) were domain pairs in the same CATH superfamily or true matches and the remaining 3114075 (3059841) were non-homologous pairs or non-matches.

The predictive ability of the models was addressed using the Nagelkerke R^2 ^measure. The R^2 ^statistic is a measure of the effect size and indicates how useful the explanatory variables are in predicting the response variable.

## Availability and requirements

Project Name: textCATH

Project Webpage: 

Operating System: Linux x86

Programming Language: Perl, Java

Other requirements: Lucene (available from )

License: GNU General Public License

## Authors' contributions

AK designed and carried out the study and wrote the paper. OCR prepared the structural and sequence comparisons for the CATH dataset. DTJ conceived and coordinated the study and participated in writing the paper. All authors read and approved the final manuscript.
